# Albinism in Africa as a public health issue

**DOI:** 10.1186/1471-2458-6-212

**Published:** 2006-08-17

**Authors:** Esther S Hong, Hajo Zeeb, Michael H Repacholi

**Affiliations:** 1 World Health Organization, Department of Protection of the Human Environment, Geneva, Switzerland and University of California, San Francisco (UCSF), School of Medicine, San Francisco, CA, USA; 2 World Health Organization, Department of Protection of the Human Environment, Geneva, Switzerland

## Abstract

**Background:**

Oculocutaneous albinism (OCA) is a genetically inherited autosomal recessive condition and OCA2, tyrosine-positive albinism, is the most prevalent type found throughout Africa. Due to the lack of melanin, people with albinism are more susceptible to the harmful effects of ultraviolet radiation exposure. This population must deal with issues such as photophobia, decreased visual acuity, extreme sun sensitivity and skin cancer. People with albinism also face social discrimination as a result of their difference in appearance. The World Health Organization is currently investigating the issues concerning this vulnerable population.

**Methods:**

Systematic electronic search of articles in PubMed concerning albinism in Africa. Furthermore, a World Health Organization (WHO) pilot survey of albinism was drafted in English, French and Portuguese, and distributed to African countries through WHO African Regional Offices (AFRO) in an attempt to gather further information on albinism.

**Results:**

Epidemiologic data on albinism, such as prevalence, were available for South Africa, Zimbabwe, Tanzania and Nigeria. Prevalences as high as 1 in 1,000 were reported for selected populations in Zimbabwe and other specific ethnic groups in Southern Africa. An overall estimate of albinism prevalences ranges from 1/5,000 – 1/15,000. In addition, both the literature review and the survey underscored the medical and social issues facing people with albinism.

**Conclusion:**

The estimated prevalence of albinism suggests the existence of tens of thousands of people living with albinism in Africa. This finding reiterates the need for increased awareness of and public health interventions for albinism in order to better address the medical, psychological and social needs of this vulnerable population.

## Background

Oculocutaneous albinism (OCA) encompasses a heterogeneous group of genetic conditions with an autosomal recessive inheritance. It is characterized by hypopigmentation of the skin, hair and eyes due to a reduced or lack of cutaneous melanin pigment production [1]. Consequently, in Africa, the affected individuals have sandy coloured hair, white chalky skin and light brown or blue eyes, making them more susceptible to the harmful effects of ultraviolet (UV) radiation.

There are two types of OCA: tyrosinase negative (OCA1) and tyrosinase positive (OCA2). In OCA1, there is little or no melanin production due to the lack of a functional tyrosinase, the critical enzyme required in the melanin biosynthetic pathway. In the more prevalent OCA2 type [1] there is some level of tyrosinase activity, thereby producing some red-yellow photomelanin pigment that gives rise to sandy coloured hair and light brown irises [1].

There is growing evidence of social discrimination and stigmatization directed towards this population [4,5]. Along with their differences in appearance, a lack of knowledge about albinism in the community leads to such stigma. For example, the etiological beliefs about albinism continue to be heavily influenced by culture and superstition, rather than genetics [6]. The goal of this review is to discuss the current knowledge on public health aspects of albinism in Africa, focusing on the epidemiology as well as medical and social issues. We also recommend further actions to alleviate this situation for the affected populations and countries. As a basis for the review we conducted a systematic literature search on albinism in Africa and conducted a survey among African WHO Member States.

## Methods

### Identification of publications

We conducted a literature search using PubMed with no date or language limitations. The search was conducted from June to July of 2005. The key terms used in this first set of searches included: *albinism, albino(s), Africa, epidemiology, population study, prevalence, health, cancer, social and psychological*.

### Inclusion and exclusion criteria

References which were not peer reviewed, did not specifically relate to Africa and/or albinism, were duplicates or focused only on basic scientific aspects of albinism, were excluded. For a small number of articles, only the abstracts were included because the full texts were unavailable.

After reviewing the remaining abstracts and full texts of our first search, we only found pertinent information for the following countries: South Africa, Zimbabwe, Tanzania, Nigeria. Therefore, we extended the search in a country-specific manner and used key words including the countries listed above, along with the term "albinism". Efforts were made to contact some of the authors who study and had published articles concerning Albinism in Africa. Additionally, a manual search of reference lists was conducted but no additional useful references were identified.

We attempted to follow the guidelines outlined by the MOOSE group [7] but since the information obtained through our study was not intended to be strictly quantitative but rather a narrative summary, we included only the pertinent points of the MOOSE criteria.

### WHO albinism information survey

A pilot questionnaire was drafted to augment the limited results of the literature search on albinism. This is shown in Appendix 1. The topics covered included data availability, medical issues, health care access, social stigmatization, economic status and community support/outreach programs. The survey was available in English, French and Portuguese and distributed via the WHO African Regional Office (AFRO) to its African Member States.

## Results

### Systematic literature review

The search yielded a total of 306 publications. 76 publications without abstract, mainly letters and commentaries, were excluded. After application of the inclusion and exclusion criteria, only 15 publications with full texts remained. These publications were chosen because they contained epidemiological information and/or data on medical/social consequences of living with albinism. The epidemiological, medical and social concerns facing this population were analysed, by country. Prevalence data by country were abstracted and entered into a table. Information on non-quantitative issues of interest for this review were abstracted in a similar way and summarized for presentation and discussion.

### Epidemiology: prevalence

Seven publications (6 cross-sectional, 1 follow-up study) contained epidemiological data on prevalence for South Africa, Zimbabwe, Tanzania and Nigeria, as shown in Table 1. The prevalence of albinism from these studies ranged from as low as 1 in 15,000 in the East Central state of Nigeria [8] to as high as 1 in 1,000 in the Tonga tribe of Zimbabwe [9]. A paper from Cameroon [10] lacked clarity in its study design making the epidemiological data difficult to interpret. Generally, albinism is considered to be a relatively common hereditary condition among the southern African populations.

#### South Africa

In 1982 Kromberg et al. studied prevalence in the ethnic groups in Soweto and Johannesburg, South Africa. 206 individuals with albinism were surveyed and the 1970 census data for Johannesburg (803,511) was used to calculate the overall prevalence of 1 in 3,900 [11]. In terms of ethnicity, the prevalence was lowest (1/4,794) among the Xhosa people and highest (1/2,041) in the Southern Sotho population. Albinism prevalences among the Swazi (1/2,716) and the Tswana (1/3,481) were slightly lower [11].

High prevalence does not seem to exclusively occur in urban regions. A prevalence of 1 in 1,515 was reported from a prospective study of congenital anomalies of liveborn neonates in Sovenga, a more rural region in Northern Transvaal, South Africa [12]. This is somewhat higher than the national rate of 1 in 3,900 [11].

#### Zimbabwe

A school-based study in Zimbabwe ascertained 157 albinism cases among 772,758 primary school pupils, giving a prevalence of 1 in 4,922. Similar results (1/4,476) were found among secondary school students. However, there was a substantial difference in the prevalence when the data were analysed by provinces: the prevalence in Matabeleland South (1/7,539) was only half of that found in Mashonaland East (1/3,843) [13].

Similar to South Africa, there was also a sizeable difference between rural and urban populations in Zimbabwe, most of which was due to Harare's high prevalence of 1 in 2,792 and 1 in 2,661 in the primary and secondary schools, respectively. Overall, urban schools, including Harare, had a prevalence of 1 in 3,268 compared to 1 in 4,694 in the rural schools [14]. As mentioned previously, an extremely high prevalence of 1 in 1,000 was reported among the small Tonga tribe, who resides in an isolated rural community of Zimbabwe [9].

#### Tanzania

Although there have been some studies addressing the health conditions of people with albinism in Tanzania, the data available on prevalence are not as extensive as for South Africa or Zimbabwe.

A study of individuals with albinism who were registered in the Tanzania Tumour Centre in Dar-es-Salaam estimated a prevalence of 1 in 1,400. However, this estimation is difficult to extrapolate to the entire population, given the data's limitation only to those individuals enrolled in the registry [17].

#### Nigeria

Nigeria's data on the epidemiology of albinism were also sparse. A prevalence of 1 in 15,000 has been reported for the East Central state through a study investigating people with albinism attending the hospital with dermatological problems. To these individuals, a questionnaire concerning demographical information such as age and gender as well as knowledge of other persons with albinism was distributed. Then, the authors expanded the investigation to educational, health and religious institutions and markets in order to reach a broader population [8]. The prevalence in this study is considerably lower than what has been reported for the other countries, but comparisons are hampered by the study design employed.

#### Regional differences: urban vs. rural

Findings of higher prevalence in urban areas may be due to various factors. Given the higher population density, it may have simply been more feasible to collect comprehensive data from urban areas. Migration to urban areas for education, health care and indoor occupations may also be contributing factors.

In addition, since many of the studies were school-based, one must consider the role of attendance. For example, urban families may have a greater ability to send their children with albinism to school. There may be more schools in urban areas that accommodate for the visually disabled and for the UV protection needs of this population. On the other hand, some families may only be able to send one or a limited number of children to school, in which case it is possible that the affected child will not be chosen in the majority of families. All of the above situations introduce bias into the prevalence estimates.

#### Regional differences: ethnicity

Ethnic differences also influence prevalence estimates. In Zimbabwe, given that the majority (83.1%) of people with albinism belonged to the Shona tribe, the diversity in the observed prevalence may be due to a founder effect or genetic drift since the Shona population have generally limited their residence to southern Africa [14]. Kromberg et al. also reported similar variations in South Africa [11].

Aside from the limited geographic mobility, consanguinity, along with other traditional marriage practices, may also be factors to consider in evaluating current and future prevalence trends of albinism [6,8,9,11].

#### Life expectancy and mortality

Several publications report a relatively greater number of individuals with albinism in the younger age groups (below 30 years). In the East Central state of Nigeria, 89% of identified people with albinism were in the age range of 0 – 30 years [8] while another study reported that 77% were under the age of 20 in the same Nigerian state [15]. A mean age of 17.8 years was reported in Soweto, South Africa [16].

Also in Cameroon, Nigeria and Tanzania, observations indicate a low number of people with albinism in age groups above 30 [8,10,17].

Whether this truly points to an increased mortality of people with albinism at a younger age (less individuals reach higher age groups) could only be evaluated on the basis of more specific mortality data. However, such data are missing.

### Medical issues

In this particular population, ocular problems are ubiquitous. In some studies, visual difficulties have been reported to occur in 100 % of people with albinism [8,15]. Due to the lack of retinal pigment required for the normal development of the visual system [15] these individuals experience photophobia, myopia and other visual problems including nystagmus and strabismus.

Aside from the visual handicaps, UV exposure is highly detrimental to the hypopigmented skin. Lack of melanin predisposes this population to severe skin damage. The majority of these lesions are in the most sun-exposed parts of the body such as the face, ears, neck and shoulders. Skin lesions include sunburns, blisters, solar elastosis/keratosis, ephelides, lentiginosis, and superficial ulcers. Ultimately, squamous cell, and less frequently basal cell, carcinomas may occur [15-18].

### Psychological and social issues

In addition to their health concerns, people with albinism must also deal with psychological and social challenges. In Nigeria, one study collected written accounts of people with albinism. These individuals stated that they tended to be more withdrawn from social situations to avoid being noticed. They were more emotionally unstable and had less assertive personalities than people without albinism. Also, they considered their society to be generally unkind and rejecting, even though they did have close friends [4].

Much of the social discrimination appears to stem from the communities' lack of education about albinism's etiology. There is limited awareness of its genetic inheritance and therefore, traditional myths and superstitions are numerous [6]. For example, some of these beliefs link albinism with (culturally unacceptable) conception during menstruation or consider albinism as a punishment from the gods for an ancestor's wrongdoing [8]. Due to this socially rooted discrimination, the quality of life of people with albinism may be compromised. For example, they are more likely to drop out of school and face more difficulty in employment and marriage compared to the rest of the population.

Furthermore, their family members may also experience discrimination from the community. In light of the traditional myths concerning albinism's etiology, mothers of affected children may be subjected to a great deal of stigma and psychological distress.

### WHO albinism pilot survey results

WHO drafted and distributed a survey (see Additional file 1) to the African WHO Member States enquiring about information on the epidemiology, medical and social issues affecting people with albinism. The survey was intended to provide a qualitative overview of these issues in the countries concerned.

The following 12 countries returned surveys: Cameroon, Congo, Equatorial Guinea, Ghana, Guinea Bissau, Mali, Mauritius, Mozambique, Niger, Rwanda, Sao Tome and Principe, and Tanzania. Respondents were either medical doctors affiliated with the Ministry of Health (Cameroon, Congo, Ghana, Tanzania) including dermatologists (Mauritius), Ministry of Health officials (Mozambique, Niger), WHO Country Officers (Guinea Bissau) or NGO personnel/other (Congo, Equatorial Guinea, Mali, Rwanda, Sao Tome and Principe).

We distributed three language versions of the WHO Pilot Survey: English, French and Portuguese. Among the survey responders, the Francophone countries included Rwanda, Niger, Mauritius, Mali, Equatorial Guinea, Congo, Cameroon. The Anglophone countries included Ghana and Tanzania and the Lusophone countries included Sao Tome and Principe, Mozambique and Guinea Bissau. The countries were divided into Francophone, Anglophone and Lusophone groups purely on (official) language grounds. There was no intent to use this grouping for comparative analyses.

Prevalence data were unavailable for the majority of the countries. A few countries did report the prevalence of albinism, but there was much variation and, in comparison to published data, the reported high estimates appeared somewhat unrealistic. Furthermore, the data sources of these estimates were not specified.

In terms of health care, survey respondents felt that much more needs to be done to address the needs of people with albinism. None of the countries, aside from Tanzania, had specialized clinics to handle the dermatological consequences of albinism. Five countries (Sao Tome and Principe, Mozambique, Mauritius, Congo and Tanzania) reported that they provide some advice on UV radiation protection for people with albinism in clinics and hospitals. Overall, however, the care was felt to be incomprehensive due to the lack of medical personnel's awareness of albinism, discrimination against people with albinism and/or a lack of resources such as sunscreens. Also, the aid was only provided if actively sought. This passive approach raises concern considering many persons with this condition may not seek regular medical attention.

Most of the surveys reported a lack of trained medical personnel. Challenges to better health care also included the barriers existing among the health workers in approaching people with albinism (lack of sensitization), lack of finances and education among this population, high cost of protective products such as sunscreens and hats/medications and social preconceptions and marginalization. Seven of the surveyed countries (Congo, Equatorial Guinea, Guinea Bissau, Mali, Niger, Sao Tome and Principe, Tanzania) reported the use of traditional medicines by people with albinism. However, the information in the survey was limited and it could not be substantiated whether people with albinism use traditional medicines differently from the general population, both in terms of frequency and rationale.

The discrimination of people with albinism is not only limited to the health care arena. The surveys report a great amount of stigmatization in schools from fellow students and teachers and even within their own families. Stigmatization stems from traditional explanations of albinism of which there are many (see above) [8]. In addition, the curiosity of their different skin colour plays a role. Most countries reported a lack of knowledge about this health condition among the general public. Survey respondents also felt that many people with albinism did not fully understand their own condition. This social discrimination was seen as an obstacle to building relationships and finding/maintaining an occupation. Therefore, most people with albinism were generally reported to be of lower, if not the lowest, economic status in their society.

In light of these difficulties, it is not surprising that many surveys reported abuse and psychological problems within this population.

However, there is some evidence of social support for people with albinism. This includes dedicated NGOs such as "SOS Albinos in Mali" and "SOS enfants vulnerables sans frontiers" in Congo. Tanzania and Congo reported that children with albinism who have visual difficulties may enroll in specialized schools for the blind. Community outreach programs exist in some surveyed countries (Congo, Cameroon and Tanzania).

## Discussion

Albinism is a disorder that affects individuals and their families medically, socially and psychologically. For some, these latter issues may be more of a burden than the actual medical complaints. While the medical issues have been studied for decades, we have tried, through this review, to shed light upon the dearth of currently available epidemiological and public health data on albinism in Africa. Given this lack of data, a prevalence range for the general population from 1/5,000 – 1/15,000 seems plausible, indicating that tens of thousands of people in southern Africa are affected. Though low in comparison with other major health problems, these figures and the even larger numbers of indirectly affected persons, qualify albinism as a public health issue deserving further attention to increase the awareness of and information about this condition.

Our survey results augment the literature review. The main focus was on health service and social issues, and responses clearly indicate a range of particular problems for people with albinism. One concern is that health care systems appear to lack responsiveness to the needs of people with albinism in most of the countries surveyed, but there are notable exceptions. In terms of social status the survey reports that people with albinism frequently are disadvantaged, and several associated factors were noted by respondents. However, also here the picture may be broader: across Africa there are numerous examples of people with albinism in high socioeconomic strata, as professionals, politicians, musicians etc. Some have used their public status to support action for people with albinism.

In terms of our survey, there are several limitations. The number of responding countries was small and due to the dissemination mechanism employed, we had little influence on the choice of actual respondents to the survey. Therefore, it is likely that not all respondents were fully aware of the scope and depth of problems facing the population of people with albinism in their country. Accordingly, we found varying degrees of detail and specificity in the responses. The responses should be seen as anecdotal rather than based on scientific studies; in this pilot survey we did not ask for specific supporting scientific evidence. Nevertheless, the survey provided some valuable information and shed light on the gaps in knowledge. These can serve as a guide for more detailed assessments and programmes in the future.

A multidisciplinary approach is recommended for future research and intervention programmes [6]. Epidemiological research should include more representative and better defined populations. The issue of premature mortality among people with albinism clearly needs to be explored further, as well as the hypothesized causes of urban/rural, regional and ethnic differences. Medically, health care providers need to be educated about albinism and the special needs of this patient population. Socially, efforts need to be made to increase awareness on the different aspects of this disorder. By informing the public about albinism, one can hope to gradually decrease the discrimination within the health care arena as well as in the society at large.

### Public health programmes and intervention recommendations

Public health programmes need to take into account the various challenges facing people with albinism. Currently, there are some programmes in place to address the medical concerns of this population in certain parts of Africa. For example, the Regional Dermatological Training Center (RDTC) in Moshi, Tanzania runs a mobile skin care clinic where a doctor and a nurse regularly visit villages to check the skin of people with albinism and provide education on protection from UV exposure [19]. Also, in South Africa, at a school for the visually impaired, there are covered walkways, trees in the courtyard and shutters on the windows in order to decrease the UV exposure [5].

However, further efforts in more African countries are required to adequately address health and social needs of people with albinism. Many of the following recommendations have been mentioned in previous publications [5,6]. Our suggestions for action include:

• Conduct research/surveys to determine the prevalence of albinism in the country

• Based on research information, develop appropriate strategies for assisting people with albinism that include the following:

 Integrate albinism awareness in the school curricula, especially to correct misconceptions about the etiology of albinism

 Educate counsellors in schools about albinism

 Train health care providers at clinics and hospitals about albinism and the effects that UV exposure can have on this condition

 Encourage community self-help support groups

 Implement programmes to aid people with albinism in finding indoor occupations

WHO's INTERSUN programme [20] provides information about the adverse health effects of excessive UV exposure and can serve as a resource for national and local authorities.

## Conclusion

Albinism, especially in Africa due to extreme sun exposure, is a condition that requires further attention than in the past. Although prevalence data are scarce and further epidemiologic research is needed, the number of people living with albinism in Africa is likely to be as high as tens of thousands. Our findings underscore the need to better address the already known medical problems facing people with albinism, but also issues of social discrimination against this population. Some progress has been made thus far in terms of medical and social care but we hope to further increase the awareness of albinism throughout African societies in the future. Public health action should focus on educational, medical and occupational settings.

## Competing interests

The author(s) declare that they have no competing interests.

## Authors' contributions

MR developed the project idea. EH and MR formulated the questionnaire. EH performed the literature review. EH and HZ organized and analysed the survey, drafted and critically reviewed the paper. MR provided sections to introduction and discussion. All authors reviewed and agreed on the final version.

## Pre-publication history

The pre-publication history for this paper can be accessed here:



## Supplementary Material

Additional File 1 Albinism: Information Survey WHO 2005. The pilot survey was drafted in English, French and Portuguese and then distributed through the WHO Regional Offices in order to gather further insight into the problems facing people with albinism and any available epidemiological information throughout Africa. Click here for file

## Figures and Tables

**Table 1:** Estimated prevalence and number of people with albinism by country

**Author**	Country/Population*	**Study Details**	**Prevalence**	Estimated Number in country**
Kromberg et al.(*11*)	South Africa/45,026,000	Cross-sectional study. Subjects were ascertained through all 120 schools, 6 health clinics and 2 hospitals in Soweto and interviewed; 213 albinism cases found; 206 interviewed	1/3,900	11,545
Venter et al. (*12*)	South Africa/45,026,000	Prospective hospital based study on liveborn neonates in Sovenga; 5 albinism cases	0.66 per 1,000 live births (Incidence)	29,720^+^
Kagore et al. (*14*)	Zimbabwe/12,835,000	Postal survey of all secondary schools within the Harare regional office area; second mailing in 3 months; visited, if there was no response	1/2,833	4,531
Lund (*13*)	Zimbabwe/12,835,000	Nationwide survey to schools; age range of 6–23 yrs; 278 albinism cases	1/4,728	2,715
Lund et al. (*9*)	Zimbabwe/12,835,000	Follow up on the above study which identified a cluster of OCA2^# ^in Tonga community; 11 albinism cases; 5 adults and 1 school girl were interviewed	1/1,000	---
Luande et al. (*17*)	Tanzania/36,977,000	Cross-sectional study. Questionnaire to 350 registered people with albinism in Tanzania Tumor Centre	1/1,400	---
Okoro (*8*)	Nigeria/124,009,000	Cross-sectional study. Questionnaires distributed to people with albinism who came to the hospital; expanded the study to educational, health, religious institutions and markets in East central state; 517 albinism cases	1/15,000	8,267

() Reference source

* Source of population data as of 2003:

** Although it is a crude estimate, we used the prevalence of the specific population in the study and the total population in the country to extrapolate the absolute number of people with albinism within a country

# OCA2 = Oculocutaneous albinism Type 2 (Tyrosine positive)

+ The absolute number in this case was estimated from a prevalence rate of 1 in 1,515 which was provided by the study's authors

--- The population studied makes it difficult to generalize the prevalence to the entire country. Therefore, the absolute number within the country was not estimated
