# Supplementary material for: Albinism in Africa as a public health issue
Source: BMC Public Health. 2006 Aug 17;6:212. doi: 10.1186/1471-2458-6-212 (PMC1584235; doi:10.1186/1471-2458-6-212)
Supplement: Additional File 1 — Albinism: Information Survey WHO 2005. The pilot survey was drafted in English, French and Portuguese and then distributed through the WHO Regional Offices in order to gather further insight into the problems facing people with albinism and any available epidemiological information throughout Africa. [file 1471-2458-6-212-S1.doc]

**ALBINISM: Information Survey – WHO 2005**

The World Health Organization (WHO) established the INTERSUN global UV program 10 years ago to assist Member States develop sound ultraviolet (UV) protection programs. Of particular importance is to ensure that vulnerable groups are protected, one of which includes the Albino population. Africa has been selected for this survey since the UV levels are generally high and albinos can be highly affected since they have little or no natural defense to UV exposure. In addition, there is some evidence that albinos may be affected socially because of their lack of pigmentation.

OBJECTIVE: Data gathered from this questionnaire will provide input for a preliminary report on the health and socio-economic concerns of the albino population in Africa and possible ways to improve the quality of life of this vulnerable group.

Thank you for your participation.

| **SECTION 1** |
| --- |

Please type in:

| **Country/Region** |  |
| --- | --- |
| **Institution** (choose code below) |  |

**1**= Country’s Ministry of Health **4**= Dermatological Society

**2**= WHO Country Office **5**= Non-governmental Organization/International Aid Organization

**3**= WHO Regional Office

| **Contact Information** |  |
| --- | --- |
| Name |  |
| Telephone Number |  |
| Fax Number |  |

SECTION 2: Please tick in the appropriate box

|  | Question | YES | NO | Please specify or explain, if applicable |
| --- | --- | --- | --- | --- |
| 1 | Is the prevalence of Albinos in your country known? If so, please specify |  |  |  |
| 2 | What is (are) the main concerns (s) facing albinos in your country? |  |  |  |
| 3 | Are there clinics/hospitals specialized & trained to treat the health problems of Albinos? If so, how many and where? |  |  |  |
| 4 | Do clinics provide advice on UV-radiation protection for Albinos? If so, examples? |  |  |  |
| 5 | Do you think it is more difficult for Albinos to receive adequate health care? If so, why or why not? Please give a brief explanation for your response. |  |  |  |
| 6 | Do Albinos use traditional medicines or healers for their medical problems? |  |  |  |
| 7 | If you answered YES to #6, do you think it is because of : |  |  |  |
| 7a | Past experience |  |  |  |
| 7b | Cost |  |  |  |
| 7c | Less Discrimination/Stigmatization |  |  |  |
| 7d | Disease severity |  |  |  |
| 7e | Does not believe in modern medicine |  |  |  |
| 8 | Is there a high incidence of psychological mental problems among Albinos? |  |  |  |

|  | Question | YES | NO | Please specify or explain, if applicable |
| --- | --- | --- | --- | --- |
| 9 | Do you think Albinos know enough about their own condition to prevent health effects? |  |  |  |

**Section 3: Pleas**e tick the appropriate box

|  | Question | YES | NO | Please specify of explain, if applicable |
| --- | --- | --- | --- | --- |
| 10 | Do Albinos experience social discrimination/stigmatization? |  |  |  |
| 10a | If you answered YES to #10, please provide examples (consider family, friends, school, work, health care, etc) |  |  |  |
| 11 | Is there a high rate of abuse towards Albinos, especially women and children? |  |  |  |
| 12 | How would you rate the economic status of most Albinos? |  |  |  |
| 12a | Upper class |  |  |  |
| 12b | Middle class |  |  |  |
| 12c | Low class |  |  |  |
| 12d | Lowest class |  |  |  |
| 13 | Do you think the disease of Albinism negatively affects their social relationships? |  |  |  |
| 14 | Are there any social support groups available for Albinos (such as finding jobs away from sunlight)? If so, please provide examples |  |  |  |
| 15 | Are there any special school in your region/country to address the needs of Albinos? If so, where? |  |  |  |
| 16 | Are there any outreach community programs to educate the public on Albinism, especially targeting parents of Albino children and teachers? If so, provide examples |  |  |  |
